# Enterovirus 71 antagonizes the inhibition of the host intrinsic antiviral factor A3G

**DOI:** 10.1093/nar/gky840

**Published:** 2018-09-21

**Authors:** Zhaolong Li, Shanshan Ning, Xing Su, Xin Liu, Hong Wang, Yue Liu, Wenwen Zheng, Baisong Zheng, Xiao-Fang Yu, Wenyan Zhang

**Affiliations:** 1 The First Hospital of Jilin University, Institute of Virology and AIDS Research, Changchun 130021, PR China; 2 Cancer Institute (Key Laboratory of Cancer Prevention and Intervention, Ministry of Education), Second Affiliated Hospital, School of Medicine, Zhejiang University, Hangzhou 310058, PR China

## Abstract

Although the host restriction factor APOBEC3G (A3G) has broad spectrum antiviral activity, whether A3G inhibits enterovirus 71 (EV71) has been unclear until now. In this study, we demonstrated for the first time that A3G could inhibit EV71 virus replication. Silencing A3G in H9 cells enhanced EV71 replication, and EV71 replication was lower in H9 cells expressing A3G than in Jurkat cells without A3G expression, indicating that the EV71 inhibition was A3G-specific. Further investigation revealed that A3G inhibited the 5′UTR activity of EV71 by competitively binding to the 5′UTR through its nucleic acid binding activity. This binding impaired the interaction between the 5′UTR and the host protein poly(C)-binding protein 1 (PCBP1), which is required for the synthesis of EV71 viral proteins and RNA. On the other hand, we found that EV71 overcame A3G suppression through its non-structural protein 2C, which induced A3G degradation through the autophagy–lysosome pathway. Our research provides new insights into the interplay mechanisms of A3G and single-stranded positive RNA viruses.

## INTRODUCTION

Enterovirus 71 (EV71), a member of the enterovirus A species of the *Picornaviridae* family, causes hand, foot and mouth disease (HFMD), which has become a severe public health problem causing both economic and social panic. Therefore, further understanding of the pathogenesis of EV71 is particularly important for treating and preventing HFMD. EV71 is capable of inhibiting innate immunity-related factors, such as the type I interferon (IFN-I), through its non-structural proteins (1–3), but whether EV71 overcomes intrinsic host antiviral factors, such as APOBEC3G (A3G), and how EV71 antagonizes the A3G protein have not been investigated until now.

EV71, which was first recognized in California in 1969 (4), is a single-stranded positive RNA virus that has approximately 7410 nucleotides with a single open reading frame (ORF) encoding a polyprotein flanked by untranslated regions (UTR) at its 5′ and 3′ ends (5). Picornavirus RNA translation is driven by the internal ribosome entry site (IRES) element located in the 5′UTR (6,7). Previous studies demonstrated that host proteins, such as hnRNP A1, hnRNP K and protein poly(C)-binding protein 1 (PCBP1), could interact with the 5′UTR of picornaviruses and promote viral protein translation and virus replication (8–13). The polyprotein can be divided into three genomic regions (P1, P2 and P3). The P1 region encodes the capsid, which comprises four structural proteins: VP1, VP2, VP3 and VP4. The P2 and P3 regions encode the non-structural proteins, including 2A, 2B, 2C, 3A, 3B, 3C and 3D (14).

In addition to innate immunity, a number of intrinsic host antiviral factors, such as A3G and SAMHD1, were discovered and identified as potential HIV-1 inhibitors (15–17). A3G, which belongs to the APOBEC3 family, has been investigated extensively and is a broad spectrum antiviral inhibitor of retroviruses, such as HIV-1; DNA viruses, such as hepatitis B virus (HBV) and human papillomavirus (HPV); single-stranded viruses, such as measles and respiratory syncytial viruses; and endogenous retro-elements, such as LINE-1 and Alu (18–23). A3G suppresses viral replication by deaminating viral cDNA cytidines to uridines or affecting viral reverse transcription or integration (17,20,24). In addition to cytidine deaminase activity, another characteristic of A3G, its nucleic acid binding activity, also contributes to its virus inhibitory function (21,25,26). However, viruses have developed sophisticated strategies to evade host antiviral factors to replicate efficiently in host cells (27–29). HIV-1 is well known to thwart this restriction through its accessory protein viral infectivity factor (Vif), which degrades the A3G protein and inactivates A3G anti-viral activity by recruiting the Cullin5-ElonginB-ElonginC-CBFβ E3 ubiquitin ligase through a proteasome pathway (30–32). Aside from the ubiquitin–proteasome pathways, the autophagy–lysosome pathway is another main route of protein and organelle clearance in eukaryotic cells (33). For instance, EV71 infection can activate autophagy and increase viral replication, with its non-structural 2C protein playing an important role in this process (34,35).

A3G has been reported to inhibit many types of viruses. However, whether A3G can suppress the replication of single-stranded positive RNA viruses, such as EV71, and whether the EV71 virus evolved to have diverse strategies for overcoming A3G restriction have not yet been investigated. In this study, we determined for the first time that ectopic A3G could inhibit EV71 viral replication in HEK293T cells infected with EV71 viruses. Moreover, EV71 replication, indicated by viral protein and RNA levels, was also far lower in H9 cells expressing endogenous A3G than in Jurkat cells that do not express the A3G protein. Further investigation revealed that the nucleic acid binding characteristic of A3G is indispensable for EV71 inhibition because it competitively binds to the 5′UTR with the host protein poly(C)-binding protein 1 (PCBP1), which is important for viral RNA synthetic and translational activities. However, EV71 developed a strategy in which the 2C protein induces A3G degradation through an autophagy–lysosome pathway to antagonize A3G restriction. The interplay mechanisms of EV71 and the intrinsic host antiviral factor APOBEC3 reported in this study not only broaden our insight into the interactions between viruses and hosts but also provide an attractive target for the development of a novel anti-EV71 inhibitor.

## MATERIALS AND METHODS

### Plasmid construction

RNA of EV71 CC063 was extracted from the supernatant of CC063 virus infected Vero cells with Trizol (Invitrogen, Carlsbad, CA, USA) and reverse transcribed with oligo (dT) primers and M-MLV reverse transcriptase (Invitrogen) according to the manufacturer's instructions (36). The resulting cDNA was used for amplification of EV71 5′UTR, 2B-HA, 2C-HA, 3A-HA, 3AB-HA, 3C-HA and 3D-HA fragments. The PCR products were subcloned into *Sal*I/*Bam*HI sites of VR1012 with N-terminal hemagglutinin (HA) tag. The pcDNA-2A-V5 plasmid was a gift from Shih-Yen Lo (Department of Laboratory Medicine and Biotechnology, Tzu Chi University, Hualien, Taiwan). Bicistronic EV71-5′UTR-luciferase-pIRIGF plasmid was constructed by amplification of EV71 5′UTR, and the fragment of 5′UTR was inserted into the *Nsi*I/*Sal*I sites of pIRIGF (Addgene, #101139). Truncated of 2C-HA and 5′UTR were constructed by amplification and inserting fragments of truncations into VR1012 vector. Site mutations of 2C-HA were constructed by site-directed mutagenesis.

The negative control vector VR1012 or the expression vectors A3A-HA, A3B-HA, A3C-HA, A3D-HA, A3G-HA, A3G-myc, A3G-V5, A3F-V5, hVif-HA, ubiquitin-flag, PCBP1-HA and A3G mutants in the 123–127 motif were described previously (32,37–39). A3G truncated 1–156 and 157–384, gift of Shan Cen, were described previously (40). hA3H-II tagged with HA was cloned into *Xho*I/*Hin*dIII sites of pcDNA3.1(–). The A3G-C291S mutant was generated by site-directed mutagenesis. Sh-A3G-pLKO.1 was constructed by annealing primers and inserting it into *Eco*RI/*Age*I sites of pLKO.1. EV71 5′UTR expression plasmid for EMSA assay was constructed by amplification and inserting fragment of 5′UTR into *XhoI/BamHI* sites of pcDNA3.1(–).

Fragment of EV71-2C was cloned into *Sal*I/*BamH*I sites of pEGFP-C1 to produce the EV71-2C-GFP expression vector. P62 expression plasmid was constructed by amplifing from HEK293T cells cDNA and inserting fragment of P62 into *Sal*I/*Bam*HI sites of VR1012 with C-terminal flag tag. A3G was amplified and cloned into *Xho*I/*Hin*dIII sites of mCherry-C1 to produce the A3G-cherry expression vector.

### Cell culture and viruses

HEK293T (ATCC catalog no. CRL-11268), Hela (ATCC catalog no. CCL-2) cells were cultured as monolayers in Dulbecco's modified Eagle's medium (DMEM) and minimum essential medium (Hyclone, Logan, UT, USA) supplemented with 10% heat-inactivated (56°C, 30 min) fetal bovine serum (FCS, GIBCO BRL, Grand Island, NY, USA) and maintained at 37°C with 5% CO_2_ in a humidified atmosphere. H9 [derivative of HuT 78] (ATCC, HTB-176) and Jurkat Clone E6-1 (ATCC, TIB-152) cells were maintained in RPMI 1640 medium (Hyclone) supplemented with 10% fetal bovine serum with penicillin/streptomycin. EV71 CC063 was isolated from HFMD patients in 2010 (41). CA16 024 virus was isolated and described by Li *et al.* (42).

### Stable A3G silenced H9 cell lines

HEK293T cells were co-transfected with Sh-A3G-pLKO.1 or pLKO.1 plus RRE, REV and VSV-G with Lipofectamine 2000 (Invitrogen). At 48 h post transfection, supernatants containing packaged lentivirus were harvested and used to infect H9 cells for 48 h. Puromycin (1.5 μg/ml) was then added into the culture to screen for stable cell lines in which A3G was shut down.

### Transfection and infection

HEK293T cells were transfected with Lipofectamine 2000 (Invitrogen) according to manufacturer's instruction, while H9 cells were nuclefected with an Amaxa human T-cell Nucleofector kit (Lonza, Switzerland) with the program U-014.

For virus infections, briefly, cells grown to 80% confluency in a six-well plate, were washed twice with phosphate-buffered saline (PBS) and incubated with virus at 37°C for 1 h. During adsorption, the plate was gently agitated at 15 min intervals. Following adsorption, the virus-containing medium was replaced with fresh medium containing 2% FCS, followed by incubation at 37°C in 5% CO_2_ for indicated time points.

### Western blotting and antibodies

Briefly, transfected or infected HEK293T, RD, H9 or Jurkat cells were harvested and boiled in 1X loading buffer (0.08 M Tris, pH 6.8, with 2.0% SDS, 10% glycerol, 0.1 M dithiothreitol and 0.2% bromophenol blue) followed by separation on a 12% polyacrylamide gel. Proteins were transferred onto a PVDF membrane for Western blot analysis. The membranes were incubated with primary antibodies, followed by a corresponding alkaline phosphatase (AP)-conjugated secondary antibody (Jackson Immunoresearch, Suffolk, UK) diluted 1:1000. Proteins were visualized using the substrates nitroblue tetrazolium (NBT) and 5-bromo-4-chloro-3-indolyl phosphate (BCIP) obtained from Sigma (St. Louis, MO, USA).

The following antibodies were used in this study: polyclonal antibody (pAb) against EV71 and CA16 was obtained from rabbits immunized with EV71 and CA16 whole viruses in our laboratory respectively, anti-hemagglutinin (anti-HA) monoclonal antibody (mAb, Covance, Princeton, NJ, USA, MMS-101R-10000), anti-tubulin mAb (Abcam, Cambridge, MA, USA, ab11323,), anti-V5 mAb (Invitrogen, R960-25), anti-myc mAb (Millipore, Billerica, MA, USA), anti-GFP pAb (Invitrogen, A-21311), anti-A3G (Cell signaling technology, 43584), anti-flag mAb (Sigma, F1804), anti-APOBEC3C rabbit pAb (Proteintech, 10591-1-AP), anti-PPIA rabbit pAb (Sangon Biotec, Shanghai, CHN, D122908), anti-ElonginC rabbit pAb (Sangon Biotec, D123299), goat anti-Mouse IgG (H+L) Highly Cross Adsorbed Secondary Antibody, Alexa Fluor Plus 488 (Invitrogen, A32723).

### RNA extraction and RT-qPCR

For RT-qPCR, viral RNA was extracted from HEK293T or RD cells transfected with APOBEC3 expression vector or cells infected with EV71 virus by Trizol reagent (Invitrogen), diethyl pyrocarbonate (DEPC)-treated water and RNase inhibitor (New England BioLabs, Ipswich, MA, USA). The cDNA was generated with a High-Capacity cDNA Reverse Transcription kit (Applied Biosystems, Carlsbad, CA, USA) and oligo d(T)18 primers according to the supplier's instructions. In order to avoid contamination, DNase was used to digest DNA (Promega, M6101). RT-qPCR was carried out on an Mx3005P instrument (Agilent Technologies, Stratagene, La Jolla, CA, USA) with the RealMaster Mix (SYBR Green Kit, Takara, Shiga, Japan) and primers designed by the conserved sequences of human A3G (hA3G). The RT-qPCR assay was carried out in a 20 μl volume consisting of 9 μl of 2.5 × RealMaster Mix/20× SYBR Green solution containing HotMaster Taq DNA Polymerase, 1 μl of 5 μmol/l of each oligonucleotide primer and 2 μg of cDNA template. Amplification of the target fragment was carried out as follows: initial activation of HotMaster Taq DNA Polymerase at 95°C for 2 min, followed by 45 cycles of 95°C for 15 s, 57°C for 15 s and 68°C for 20 s.

### Luciferase assays

HEK293T cells in 12-well plates were co-transfected with 0.5 μg bicistronic pIRIGF-5′UTR expression vector and indicated A3 protein for 48 h before harvesting. Luciferase activity was detected in the cells with Fluoroskan Ascent FL (Thermo) with Dual-Luciferase Reporter Assay System (Promega).

### Co-immunoprecipitation (co-IP)

Co-IP experiments were performed as previously reported (32,39). For EV71 2C-HA IP, HEK293T cells transfected with hA3G-V5 and 2C-HA or Vif-HA were treated with Bafilomycin A1 (Baf-A1, Millipore, Billerica, MA, USA) for another 12 h prior to harvest. The cells were then harvested and washed twice with cold PBS, followed by disruption with lysis buffer (PBS containing 1% Triton X-100 and complete protease inhibitor cocktail [Roche]) at 4°C for 1 h. Cell lysates were clarified by centrifugation at 10 000 × *g* for 30 min at 4°C. Anti-HA agarose beads (Roche, Basel, Switzerland, 190–119) were mixed with the pre-cleared cell lysates and incubated at 4°C for 4 h on an end-over-end rocker. The reaction mixtures were then washed six times with cold wash buffer (20 mM Tris–HCl, pH 7.5, 100 mM NaCl, 0.1 mM EDTA, 0.05% Tween-20) and subsequently analyzed by immunoblotting. For A3G or PCBP1 with 5′UTR IP, the reaction mixtures were washed eight times with lysis buffer and subsequently analyzed by immunoblotting or extracted RNA for RT-qPCR analysis.

### Confocal microscopy

For 2C and A3G, Hela cells were transfected with 2C-GFP and A3G-cherry for 48 h, for the localization of p62 and A3G in the presence or absence of EV71 2C, p62-Flag and A3G-Cherry plus VR1012 or 2C-HA were cotransfected into Hela cells. The cells were treated with 10 nM Baf-A1, *Streptomyces griseus* for another 12 h prior to fixing, then fixed in 4% paraformaldehyde at room temperature for 15 min, washed with PBS, permeabilized in 0.1% Triton X-100 for 5 min, washed in PBS, blocked in 2% BSA for 1 h, and then incubated at room temperature for 2 h with mouse anti-flag antibody (sigma) at 1:1000. Following a wash, cells were incubated with Goat anti-Mouse IgG at room temperature for 1 h. After being washed with cold PBS, cells were analyzed by using a laser scanning confocal microscope (LSM710, Carl Zeiss, Oberkochen, Germany).

### Electrophoretic mobility shift assay (EMSA) and RNA pull down assay

RNA of EV71-5′UTR was transcribed with a MEGAscriptTM T7 kit (Ambion, Austin, TX, USA), and RNA purified with a MEGA clear kit (Ambion) was annealed with the DNA probe (5′-GTTTAGCTGTGTTAAGGGTCAAG-3′) labeled biotin by a Biotin 3′ End DNA Labeling Kit (Thermo). For EMSA assay, A3G and its mutants with a HA tag were purified by co-IP with anti-HA agarose beads from 5 is × 10^6^ HEK293T cells transfected with A3G or mutant expression plasmid for 48 h, and then incubated with 4 ug biotinylated RNA for the detection of the interaction between EV71-5′UTR and A3G or its mutants with a LightShift^®^ Chemiluminescent EMSA Kit (Thermo) according to manufacturer's instructions. RNA pull down assay was performed as reported previously (10,43). To be specific, 4 ug biotinylated RNA were heated to 90°C for 2 min to disrupt the secondary structure, and placed on ice for 2 min to form the proper secondary structure for EMSA and pull down assays. The 5 × 10^6^ cells were lysed in lysis buffer described in Co-IP assay supplemented with 20 U Protector RNase inhibitor (Roche), and centrifuged to get the clear cell lysate. Then the lysate were precleared with 50ul Streptavidin-Sepharose Beads (BioVision, Milpitas, CA, USA) by rotating at 4°C for 30 min. Then folded RNA were added to the precleared cell lysate and rotated at room temperature for 1 h. 100 ul beads were added to the reaction and rotated at 4°C for 1 h followed by five washes using the wash buffer described in Co-IP assay supplemented with 20 U Protector RNase inhibitor (Roche). The proteins on beads were detected by western blotting.

### Cytotoxicity assay

The cytotoxicity assay was performed with Cell Counting Kit-8 (Transgen, China). sh-A3G H9 or control pLKO.1 H9 cells seeded on 24-well plates were infected with an MOI of 1.0 for indicated time points, then cells were re-seeded onto a 96-well plate to assess according to the manufacturer's protocol. Absorbance at 450 nm was recorded using an iMark microplate reader (Bio-rad, Hercules, CA, USA).

## RESULTS

### A3G inhibits EV71 replication in HEK293T cells

**Figure 1.**
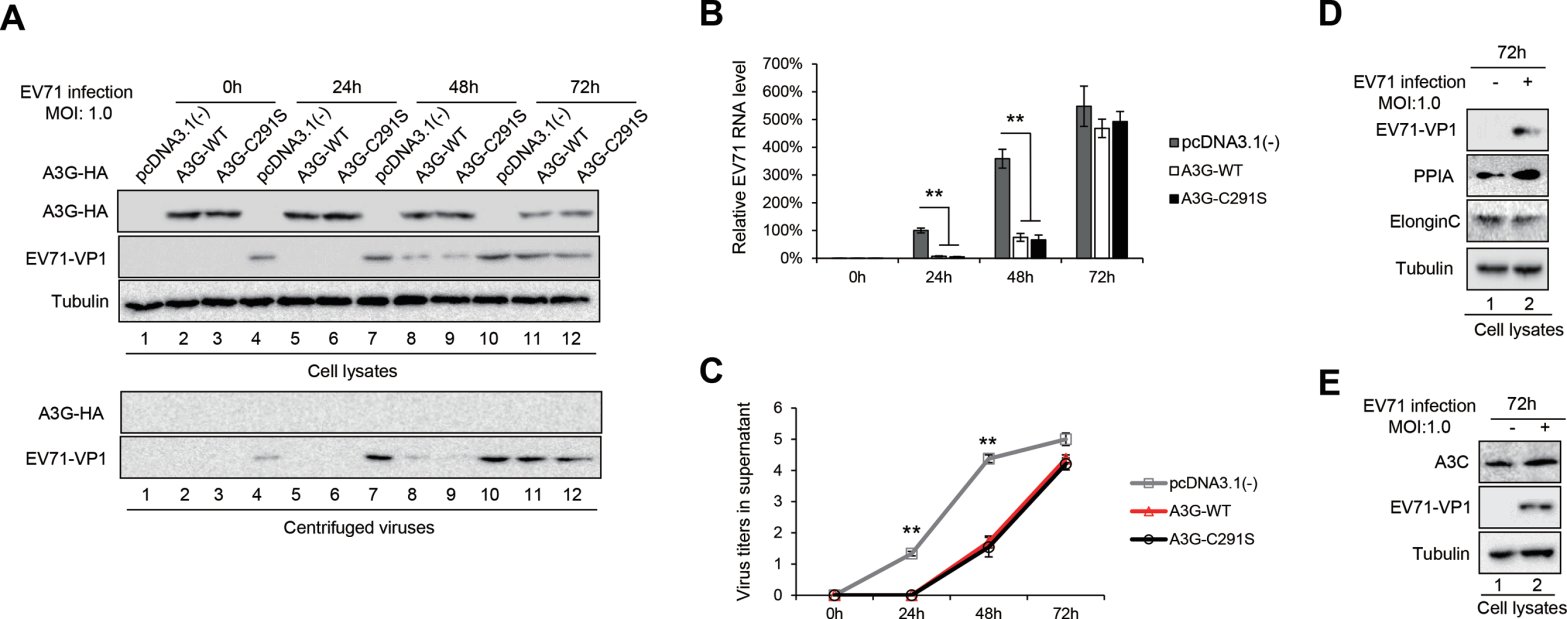
A3G and the A3G C291S mutant both inhibit EV71 replication in HEK293T cells infected with EV71 virus. HEK293T cells were transfected with pcDNA3.1, A3G-myc or A3G C291S-myc and then infected with EV71 virus at an MOI of 1.0 at 24 h post-transfection. The cells and supernatants were harvested at 24 h, 48 h and 72 h post-infection. (**A**) A3G and viral VP1 levels in the cells and supernatants were detected by immunoblotting analyses using anti-VP1, anti-myc and anti-tubulin antibodies. The supernatants from transfected HEK293T cells were concentrated using 25% sucrose prior to immunoblotting analysis. (**B**) EV71 RNA levels in cells were detected by RT-qPCR. GAPDH was used as a control. EV71 RNA levels of cells transfected with pcDNA3.1 for 24 h were set as 100%. (**C**) Viral titres in the supernatants were determined by the cytopathic effect method. The results are the means with SD from three independent experiments. The asterisks indicate statistically significant differences between groups as assessed by Student's *t*-test (**P < 0.01). The endogenous expression levels of PPIA or ElonginC in HEK293T cells (**D**) and A3C in Jurkat cells (**E**) were detected by immunoblotting analyses at 72 h post-infection.

To investigate whether A3G inhibits EV71 replication, we transfected A3G or its mutant C291S, which inactivates the cytidine deaminase of A3G (44), into HEK293T cells for 24 h and infected them with EV71 at a multiplicity of infection (MOI) of 1.0. We observed that the overexpression of A3G obviously inhibited EV71 viral replication at 24 h and 48 h post-infection (Figure 1A, lanes 5 and 8 compared to 4 and 7). Importantly, A3G C291S with a mutation at the C-terminus, which provides the cytidine deaminase activity of A3G, showed similar inhibitory effects on EV71 replication (Figure 1A, lanes 6 and 9). A3G and A3G C291S also inhibited virus production according to the supernatant Western blotting analysis (Figure 1A, lower panel, lanes 5 and 6, lanes 8 and 9) and viral RNA synthesis according to viral RNA detection using real-time quantitative PCR (RT-qPCR) (Figure 1B). Accordingly, virus titres were obviously lower in the presence of A3G or A3G C291S than in the absence of A3G (Figure 1C). Interestingly, we observed that the expression of A3G was reduced with EV71 replication, especially at 72 h (Figure 1A, lanes 11 and 12 compared to 2 and 3), which was consistent with the phenomenon that viral VP1 protein and viral RNA levels were restored at 72 h, indicating that EV71 may have developed strategies to specifically antagonize A3G restriction. Under the same conditions, the endogenous proteins PPIA and ElonginC in HEK293T cells and another restriction factor, A3C, expressed in Jurkat cells were not affected by EV71 infection (Figure 1D and E), excluding the possibility that EV71 blocked cellular proteins. Moreover, unlike the requirement that A3G be incorporated into virions to inhibit the Vif-deficient HIV-1 virus (45,46), we did not observe that A3G was packaged into EV71 virions (Figure 1A, lower panel). These results showed that A3G inhibits EV71 replication without requiring cytidine deaminase and that EV71 antagonizes A3G by reducing its expression.

### Endogenous A3G restricts EV71 replication

**Figure 2.**
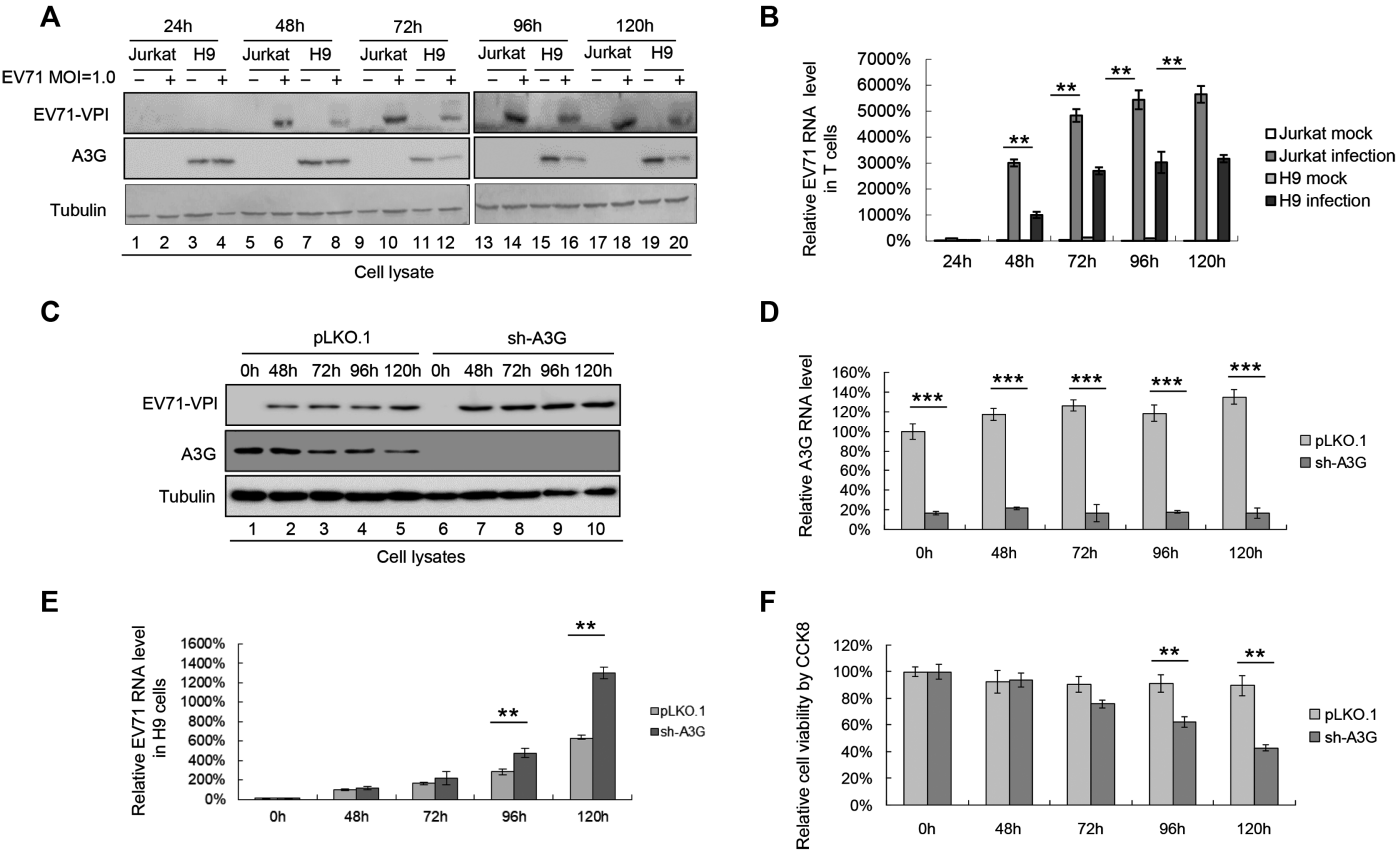
H9 cells expressing A3G have lower EV71 replication capacity than Jurkat cells without A3G expression. (**A** and **B**) EV71 replication was lower in H9 cells than in Jurkat cells. H9 and Jurkat cells were infected with DMEM or EV71 virus at an MOI of 1.0. The infected cells were harvested at the indicated time points post-infection. (A) A3G and viral VP1 levels in cells were detected by immunoblotting analyses using anti-VP1, anti-A3G and anti-tubulin antibodies. (B) EV71 RNA levels were lower in H9 cells than in Jurkat cells according to RT-qPCR detection. EV71 RNA levels of Jurkat cells infected with EV71 for 24 h were set as 100%. (C–F) Silencing A3G in H9 cells enhanced EV7 replication. H9 cells stably expressing A3G shRNA were established. A3G protein (**C**) and mRNA (**D**) levels at different time points are shown. (**E**) EV71 RNA levels were higher in A3G knockdown H9 cells than in negative control pLKO.1 cells at all time points. EV71 RNA levels of negative control pLKO.1 cells infected with EV71 for 48 h were set as 100%. (**F**) Cytotoxicity induced by EV71 in A3G knockdown H9 cells and control pLKO.1 cells was detected by CCK8 assays. Uninfected H9 cells at 0 h were set as 100%. (B, D–F) The results are the means with SD from three independent experiments. The asterisks indicate statistically significant differences between groups as assessed by Student's *t*-test (*P < 0.05, **P < 0.01, ***P < 0.001).

To confirm that A3G inhibits EV71 replication under more natural conditions, we compared the replication capacity of EV71 in the H9 cell line expressing A3G and the Jurkat cell line without A3G expression (47,48). H9 and Jurkat cells were infected with EV71 virus at a dose of 1.0 MOI and harvested at various time points. We observed that EV71 VP1 expression was generally lower in H9 cells than in Jurkat cells within 72 h (Figure 2A, lanes 6 and 8, 10 and 12, 14 and 16), indicating lower viral replication in H9 cells than in Jurkat cells. Consistent with the above observation, the RNA level of EV71 was obviously lower in H9 cells than in Jurkat cells at all indicated time points (Figure 2B), further confirming that A3G in H9 cells could inhibit EV71 viral replication. However, 96 h later, the amount of EV71 VP1 in H9 cells increased to a similar level to that observed in Jurkat cells (Figure 2A. lanes 18 and 20). We speculated that the amount of EV71 virus produced 96 h later was sufficient to antagonize the A3G restriction. The reduced A3G in EV71-infected H9 cells compared to that in uninfected H9 cells further confirmed our speculation (Figure 2A, lanes 11 and 12, 15 and 16, 19 and 20). Therefore, we deduced that EV71 might overcome A3G restriction.

To validate A3G as the main factor inhibiting EV71 replication in H9 cells, a stable cell line in which A3G was knocked down using a lentivirus system was constructed. Western blotting and RT-qPCR assays showed that the protein expression and mRNA levels of A3G were far lower in the sh-A3G-treated group than in the negative control pLKO.1 group (Figure 2C and D). A3G-silenced H9 cells (sh-A3G) and control pLKO.1 H9 cells were infected with EV71 virus at an MOI of 1.0. As the time increased, especially after 72 h, the RNA level of EV71 in the A3G-silenced group increased gradually compared to that in the negative control pLKO.1 group (Figure 2E). Concordantly, cell viability was consistently lower in sh-A3G H9 cells infected with EV71 than in pLKO.1 H9 cells infected with EV71 according to Cell Counting Kit-8 (CCK8) assay (Figure 2F). Taken together, silencing A3G in H9 cells enhances EV71 replication, and A3G plays an important role in inhibiting EV71 viral replication.

In addition to EV71, we also detected whether A3G inhibits CA16, another circulating virus causing HFMD. Overexpressing A3G in HEK293T cells restricted CA16 viral replication at 40 h post-infection according to CA16 VP1 immunoblotting and viral mRNA analyses (Supplemental Figures S1A and S1B).

### A3G restricts 5′UTR replication capacity by competitively binding to the 5′UTR with PCBP1

**Figure 3.**
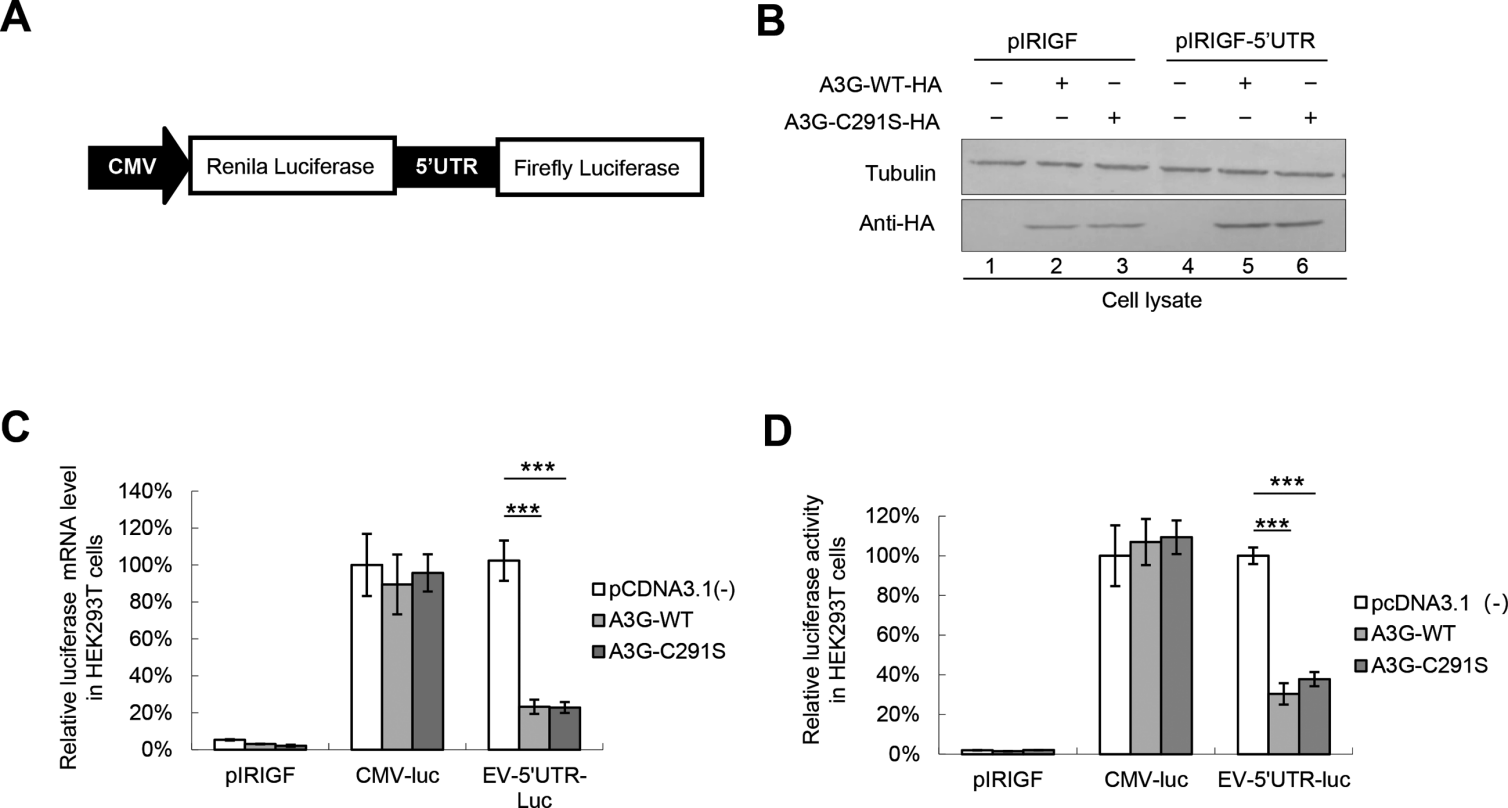
A3G inhibits EV71 5′UTR activity. (**A**) Bicistronic plasmid construction. (B–D) pcDNA3.1, A3G or A3G C291S plus the pIRIGF negative vector or bicistronic pIRIGF-5′UTR expression plasmid were co-transfected into HEK293T cells, which were harvested at 48 h post-transfection. (**B**) A3G and A3G C291S expression was detected by immunoblotting analysis. (**C**) Effects of A3G and A3G C291S on luciferase mRNA levels according to RT-qPCR analysis. GAPDH was used as a control. mRNA levels of luciferase downstream of CMV in the absence of A3G were set as 100%. (**D**) Effects of A3G and A3G C291S on luciferase activity. Luciferase activity downstream of CMV in the absence of A3G was set as 100%. (C and D) The results are the means with SD from at least three independent experiments. The asterisks indicate statistically significant differences between groups as assessed by Student's *t*-test (***P < 0.001).

Accumulating evidence has demonstrated that the IRES located in the 5′UTR of EVs is responsible for virus translation and replication (49,50); thus, we investigated whether A3G could affect 5′UTR activity. A bicistronic plasmid was constructed and used (Figure 3A). Western blotting assays showed that A3G and A3G C291S were expressed efficiently (Figure 3B). The luciferase assay showed that A3G and A3G C291S both decreased the mRNA and protein expression of luciferase downstream of the 5′UTR but not luciferase expression downstream of the CMV promoter (Figure 3C and D), indicating that A3G specifically affected the activity of the EV71 5′UTR. Consistent with the inhibition of CA16 replication by A3G, we also observed that A3G inhibited CA16 5′UTR activity (Supplemental Figures S1C and S1D), possibly due to 84.09% sequence homology of the full-length 5′UTR between EV71 and CA16. In addition, A3G specifically inhibited the 5′UTR activity of poliovirus (PV), which has 70.71% homology with the 5′UTR of EV71 (Supplemental Figures S1E and S1F).

**Figure 4.**
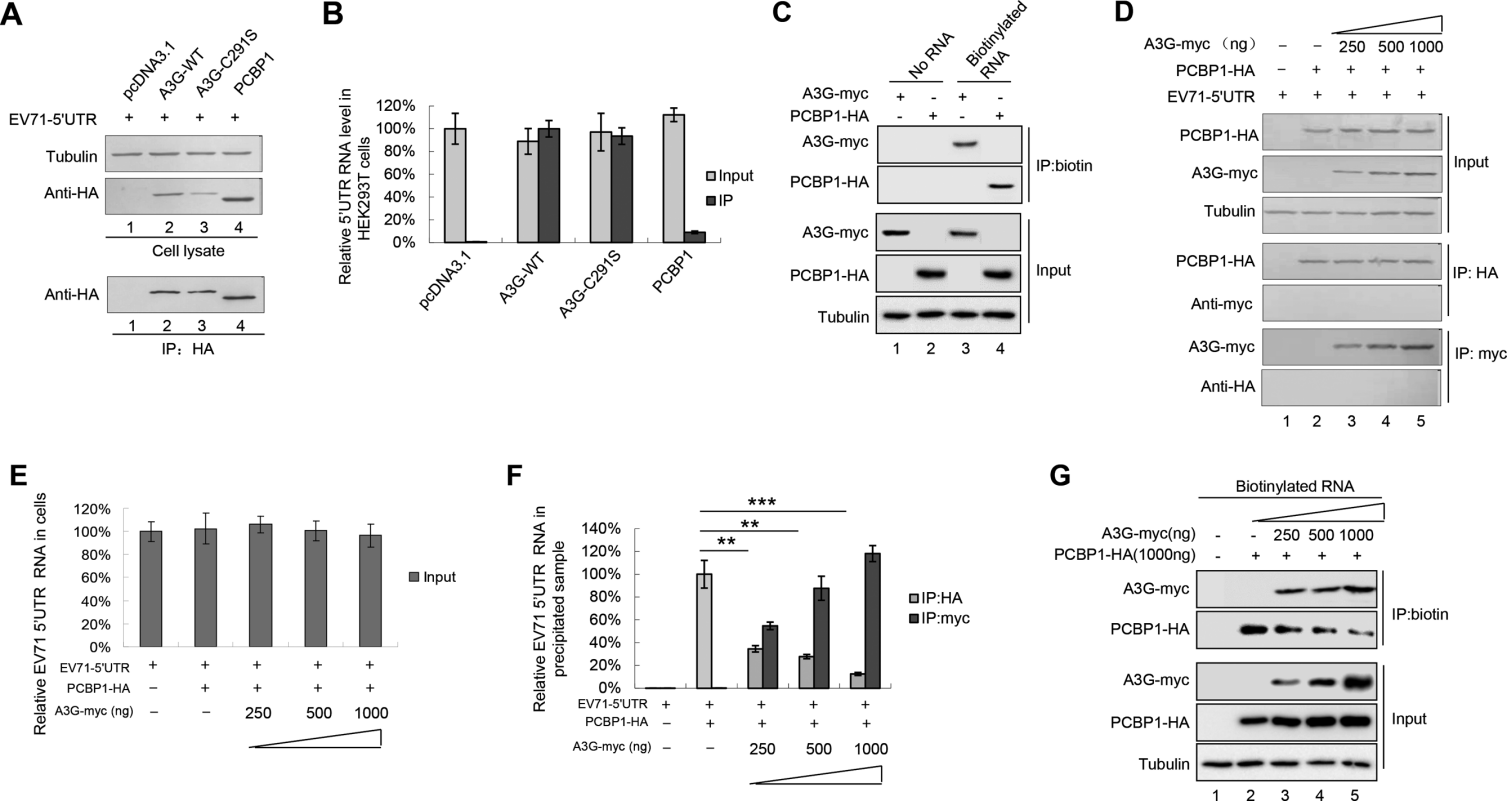
A3G competitively binds to the EV71 5′UTR with PCBP1. (**A** and **B**) A3G and PCBP1 expression was detected by immunoblotting analysis. pcDNA3.1, A3G-HA, A3G C291S-HA or PCBP1-HA was co-transfected with the 5′UTR expression vector into HEK293T cells. Cell lysates were prepared at 48 h post-transfection. Part of the cell lysates were immunoprecipitated with anti-HA agarose beads. (B) Binding capacity of A3G or PCBP1 to the EV71 5′UTR. The results are the means with SD from at least three independent experiments. (**C**) The interaction between A3G or PCBP1 with the 5′UTR of EV71 according to RNA pull-down assay. (D–F) Competitive binding assay using immunoprecipitation. HEK293T cells were transfected with increasing doses of A3G-myc and PCBP1-HA plus the 5′UTR. At 48 h post-transfection, half of the cells were harvested and immunoprecipitated with anti-HA agarose beads, and the other cells were precipitated with anti-myc agarose beads. The cell lysates and immunoprecipitated products were analysed by immunoblotting (**D**) and RT-qPCR analyses. (**E**) 5′UTR RNA input in cell lysates. GAPDH was used as a control. (**F**) Increasing amounts of A3G disrupted the interaction of PCBP1 with the EV71 5′UTR. The binding between the 5′UTR and PCBP1 in the absence of A3G was set as 100%. The results are the means with SD from at least three independent experiments. The asterisks indicate statistically significant differences between groups as assessed by Student's *t*-test (*P < 0.05, **P < 0.01, ***P < 0.001). (**G**) Increasing amounts of A3G decreased the interaction of PCBP1 with the 5′UTR of EV71 according to RNA pull-down assay.

Previous studies showed that the 5′UTR forms nucleoprotein complexes containing poly(C)-binding protein 1 (PCBP1), which is required for the replication of picornaviruses, including enteroviruses (11,37,51–53). Here, we determined whether A3G or A3G C291S binds to the 5′UTR as well as its binding ability; PCBP1 was used as positive control. A3G and A3G C291S had stronger binding ability to the 5′UTR than PCBP1 according to immunoprecipitation and RT-qPCR assays (Figure 4B), while the amount of proteins immunoprecipitated by HA beads was almost equal (Figure 4A). Similarity, the biotinylated EV71 5′UTR could pull down A3G and PCBP1 (Figure 4C). We speculated that A3G may competitively bind to the 5′UTR, which impairs the interaction between the 5′UTR and PCBP1. To verify this hypothesis, we co-transfected a 5′UTR expression vector plus pcDNA3.1 or increasing doses of A3G-myc tagged and PCBP1-HA-tagged expression vectors into HEK293T cells as indicated in Figure 4D. As expected, A3G-myc and PCBP1-HA were expressed efficiently and could be immunoprecipitated from the cell lysates (Figure 4D); 5′UTR RNA levels in the cell lysates from all samples were similar (Figure 4E). However, the interaction of PCBP1 with the 5′UTR was profoundly reduced with increasing A3G expression levels. Moreover, the increasing levels of A3G bound increasing amounts of 5′UTR (Figure 4F), which were also confirmed by RNA pull-down assays (Figure 4G). The 5′UTR did not co-precipitate with the sample in the absence of A3G-myc or PCBP1-HA, indicating that the interaction between the 5′UTR and the protein was specific (Figure 4F).

### The nucleic acid binding activity of A3G is required for its inhibition of EV71 replication

**Figure 5.**
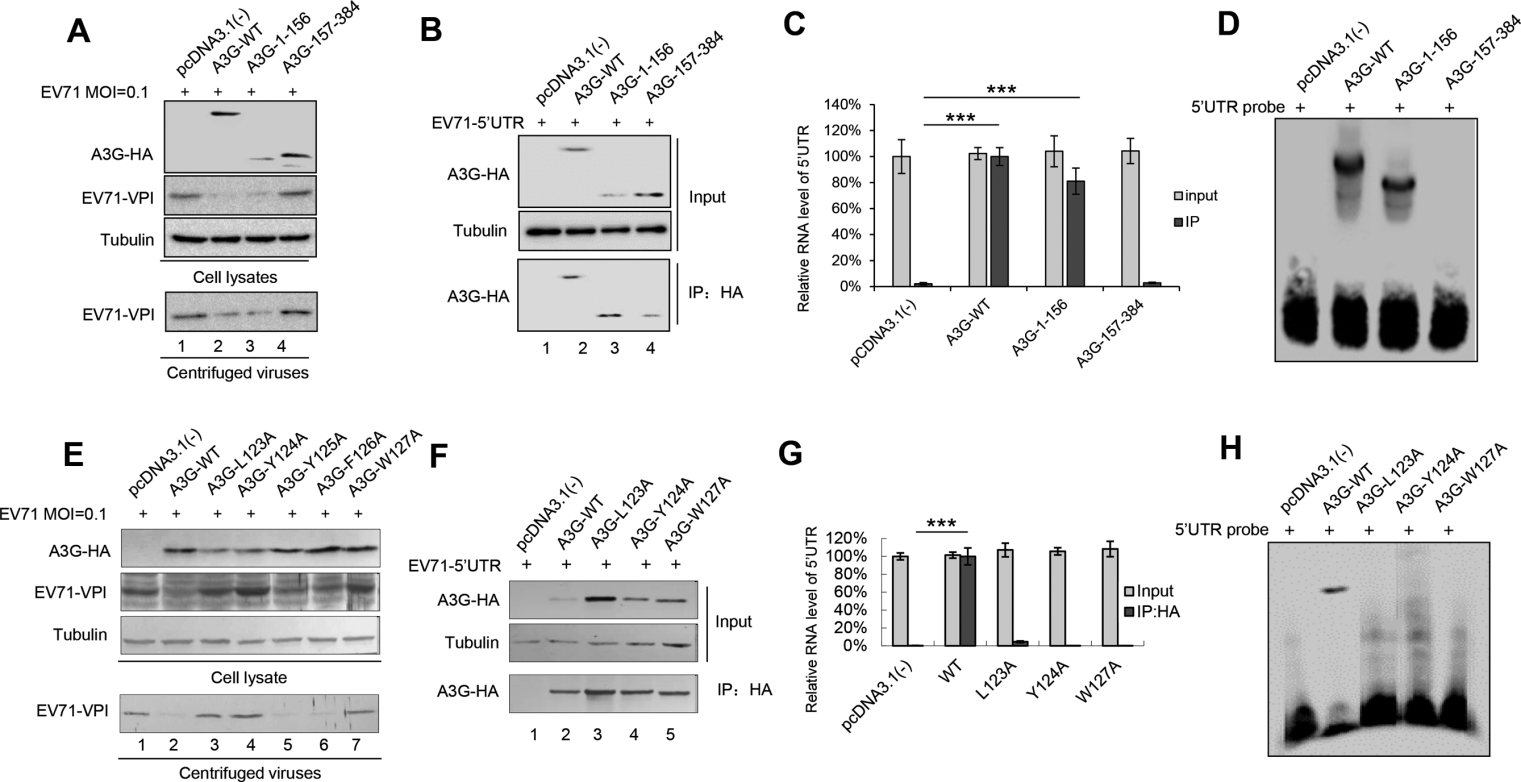
The RNA-binding property of A3G is required for its binding to the EV71 5′UTR and EV71 inhibition. (**A**) The amino-terminal domain of A3G alone could inhibit EV71 replication, but the carboxy-terminal could not. HEK293T cells were transfected with pcDNA3.1(–), A3G or the indicated mutants. At 24 h post-transfection, HEK293T cells were infected with EV71 at an MOI of 0.1. At 72 h post-infection, the cells were harvested and loaded for immunoblotting analyses using anti-VP1, anti-HA and anti-tubulin antibodies. The amino-terminal of A3G alone could inhibit EV71 replication. (**B**) Immunoprecipitation assay. HEK293T cells were transfected with A3G-HA or the indicated mutants plus the 5′UTR expression vector. At 48 h post-transfection, the cells were harvested and immunoprecipitated with anti-HA agarose beads. The cell lysates and immunoprecipitated products were analysed by immunoblotting. (**C**) The amino-terminal domain of A3G lost the ability to interact with the 5′UTR according to RT-qPCR analysis. GAPDH was used as a control. (**D**) EMSA of the EV71 5′UTR and A3G or its mutants. (**E**) A3G mutants L123A, Y124A and W127A could not inhibit EV71 replication. The assay was performed as described for panel A. (**F**) Immunoprecipitation assays were performed as indicated for panel B. (**G**) A3G mutants L123A, Y124A and W127A lost the ability to interact with the 5′UTR according to RT-qPCR analysis. GAPDH was used as a control. (**H**) EMSA of the EV71 5′UTR and A3G or its mutants. (C and G) The results are the means with SD from three independent experiments. The asterisks indicate statistically significant differences between groups as assessed by Student's *t*-test (***P < 0.001).

A3G has two domains: the carboxy-terminal domain responsible for deamination and the amino-terminal domain without catalytic activity that mediates incorporation into viral particles via its RNA binding property (8,26,54–56). Since cytidine deaminase was not associated with the anti-EV71 function (Figures 1, 3 and 4), we investigated whether the nucleic acid binding activity of A3G, another characteristic, contributes to its anti-EV71 activity. The results showed that the amino-terminal domain of wild type (WT) A3G potentially inhibited EV71 replication, but the carboxy-terminal domain could not (Figure 5A). Accordingly, co-IP and RNA EMSA confirmed that the amino-terminal domain of A3G could interact with the EV71 5′UTR, but the carboxy-terminal domain could not (Figure 5B–D). The 123–127 motif at the amino-terminus of A3G is involved in its nucleic acid binding activity (39,57); thus, we evaluated whether a single mutant in this motif affects its anti-EV71 activity. We found that amino acid mutants L123A, Y124A and W127A of A3G could not inhibit EV71 replication compared to WT A3G (Figure 5E). Immunoprecipitation and RNA EMSA further confirmed that these three mutants lost the ability to bind the EV71 5′UTR (Figure 5F–H). In addition, according to 5′UTR activity assays, we confirmed that the carboxy-terminal domain as well as L123A, Y124A and W127A mutants of A3G lost the ability to inhibit EV71 5′UTR activity compared to WT A3G (Supplementary Figure S2). These data demonstrated that the nucleic acid binding property of A3G was closely associated with its inhibition of EV71 replication.

### Loop I and loop II in the 5′UTR are required for A3G binding

**Figure 6.**
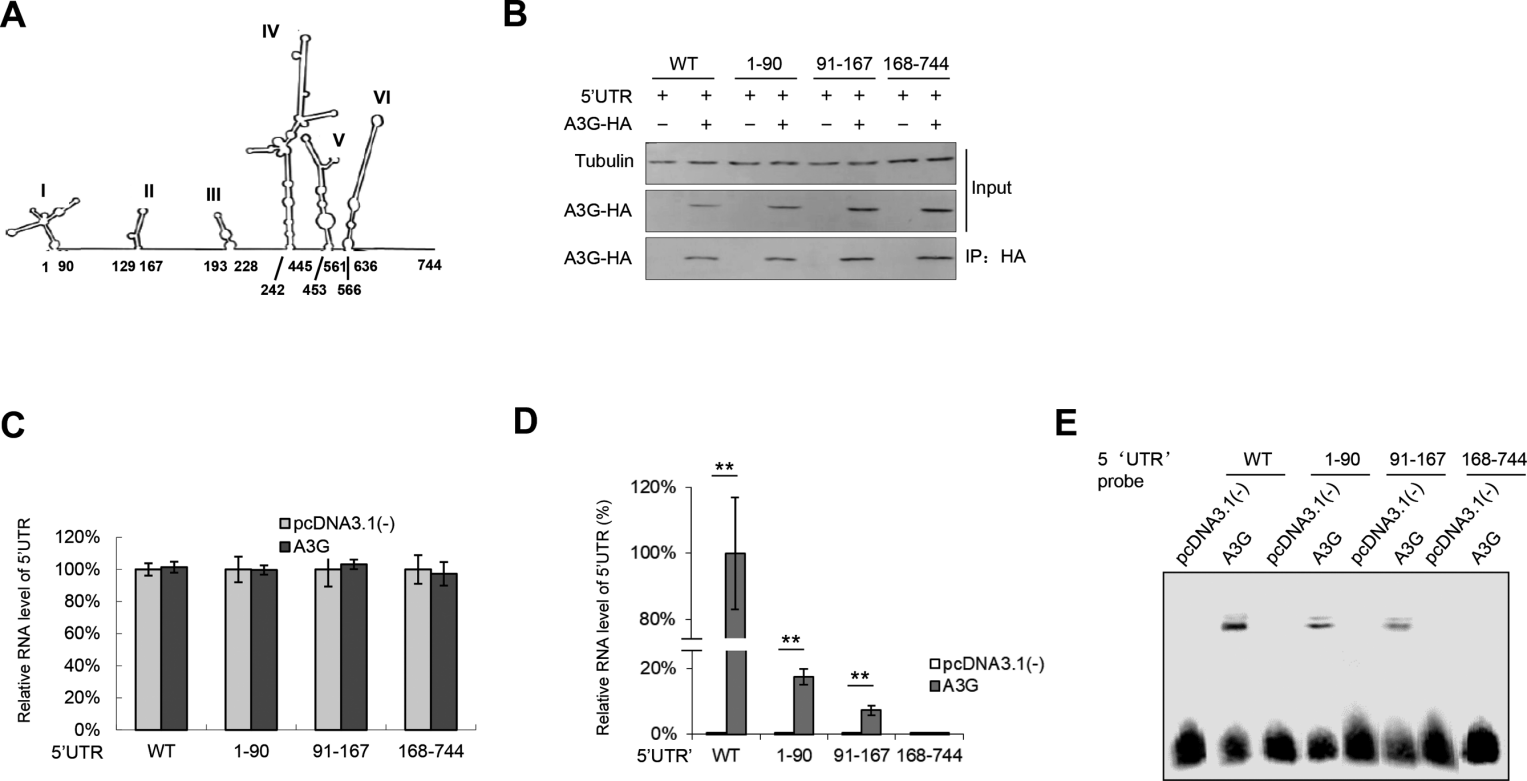
(**A**) The secondary structure of the 5′UTR was predicted by MFold. (B–D) The interactions between A3G and the 5′UTR truncated mutants. A3G or VR1012 and the WT 5′UTR or indicated 5′UTR truncations were transfected into HEK293T cells; then, the cells were harvested for IP and RT-PCR analysis at 48 h post-transfection. (**B**) A3G protein levels in the cell lysates and co-IP elutes were confirmed by immunoblotting analysis. (**C**) 5′UTR RNA input in the cell lysates was detected by RT-qPCR. (**D**) Loop I and loop II in the EV71 5′UTR maintained binding ability with A3G according to RT-qPCR detection. The RNA level of the WT 5′UTR binding to A3G was set as 100%. The results are the means with SD from three independent experiments. The asterisks indicate statistically significant differences between groups as assessed by Student's t-test (*P < 0.05, **P < 0.01, ***P < 0.001). (**E**) EMSA of the EV71 5′UTR or its truncated mutants with A3G.

To determine the binding domain in the 5′UTR required for A3G binding, we constructed three truncations of the EV71 5′UTR based on the secondary structure predicted with Mfold (Figure 6A) (9,11). Immunoprecipitation assays showed that regions 1–90 (loop I) and 91–167 (loop II) of the EV71 5′UTR maintained a certain degree of binding with A3G, but 167–744 (loops III-VI) completely lost this ability (Figure 6B–D), which was also confirmed by EMSA (Figure 6E). Coincidently, a previous study showed that loops I and IV in the 5′UTR are also required for PCBP1 binding (11), further confirming our hypothesis that A3G binds competitively to the 5′UTR with PCBP1 through its RNA-binding property to inhibit EV71 replication.

### A3A, A3D and A3F but not A3B, A3C or A3H-II inhibit EV71 replication

APOBEC3 proteins contain seven family members, including A3A, A3B, A3C, A3D, A3G, A3F and A3H, which have different levels of anti-HIV-1 activity (58). To further investigate whether other APOBEC3 proteins have the capacity to inhibit EV71 replication similar to A3G, we detected the effects of the A3A, A3B, A3C, A3D, A3F and A3H-II proteins (which have stronger anti-HIV-1 activity) on EV71 5′UTR activity. Western blotting assays showed that all of these APOBEC3 proteins were expressed efficiently (Supplementary Figure S3A). However, A3A, A3D and A3F, but not A3B, A3C or A3H-II, inhibited EV71 5′UTR activity, and these effects were slightly weaker than that of A3G (Supplementary Figure S3B). We next detected whether these APOBEC3 proteins could inhibit EV71 replication and whether they could be correspondingly downregulated by the EV71 virus. Consistent with the data showing that APOBEC3 inhibited EV71 5′UTR activity, A3A, A3D and A3F modestly inhibited EV71 replication at 72 h (Supplementary Figures S4A–E), while A3B, A3C and A3H-II showed no such effect (Supplementary Figure S4B, C and F). At 96 h post-infection, no inhibition was observed, possibly due to the breakthrough of EV71 to A3G.

### EV71 2C protein reduces the expression of A3G through autophagy–lysosome but not proteasome pathway

**Figure 7.**
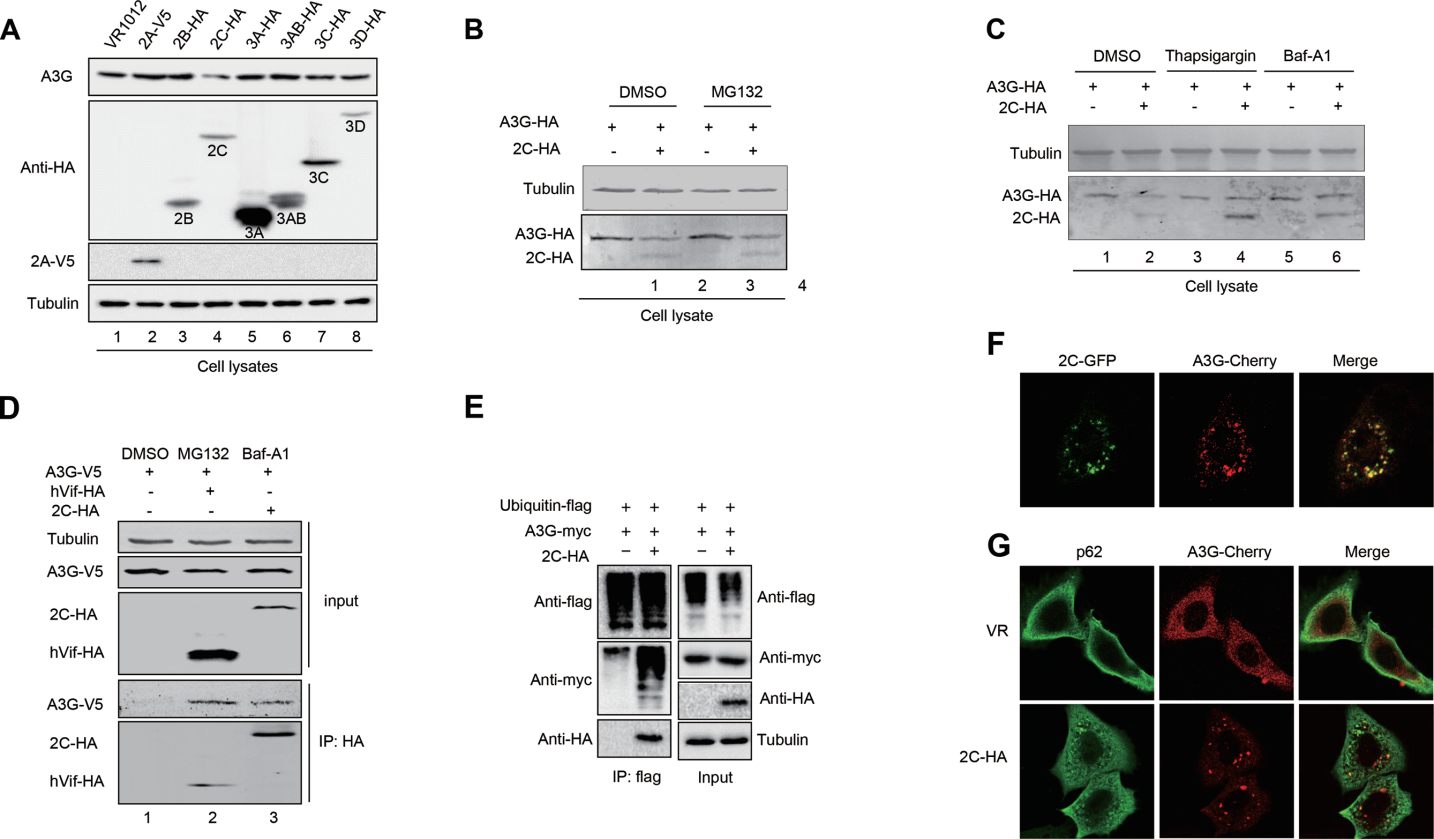
EV71 2C antagonizes A3G via the autophagy–lysosome degradation pathway but not the proteasome pathway. (**A**) 2C reduced the expression of A3G. A3G-HA plus VR1012 (negative control) or the indicated EV71 non-structural proteins were co-transfected into H9 cells. The cells were harvested for immunoblotting analysis at 48 h post-transfection. (**B**) The MG132 inhibitor could not rescue EV71 2C-mediated A3G degradation. The cells were treated for another 12 h with 10 μM DMSO or MG132 prior to harvest for immunoblotting analysis at 36 h post-transfection. (**C**) An autophagy inhibitor could rescue EV71 2C-mediated A3G degradation. The cells were treated for another 12 h with 3 μM DMSO, 3 μM thapsigargin or 10 nM Baf-A1 prior to harvest for immunoblotting analysis at 36 h post-transfection. (**D**) EV71 2C interacted with A3G according to immunoprecipitation assay. HIV-1 Vif-HA was used as a positive control. The cells were treated for another 12 h with 10 μM DMSO, 10 μM MG132 or 10 nM Baf-A1 to avoid A3G degradation as indicated prior to harvest. (**E**) A3G was ubiquitinated by EV71 2C. (**F**) Co-localization of EV71 2C and A3G in HeLa cells. 2C formed autophagic puncta and co-localized with A3G. (**G**) Co-localization of A3G and p62 in the presence or absence of EV71 2C. (F and G) Images were taken under a Zeiss LZM710 confocal microscope.

The above data showed that EV71 could reduce the expression of APOBEC3 proteins (Figures 1, 2 and Supplemental Figure S1); thus, we next examined which EV71 proteins support this function. Only 2C, not 2A, 2B, 3A, 3AB, 3C or 3D, could reduce endogenous A3G expression in H9 cells (Figure 7A). Moreover, the effect of EV71 2C on A3G occurred at the protein level but not the mRNA level (data not shown).

The ubiquitin–proteasome and autophagy–lysosome pathways are the two main routes of protein and organelle clearance in eukaryotic cells (33). We used the proteasome inhibitor MG132 and the autophagy–lysosome inhibitors thapsigargin and Baf-A1 to further investigate the mechanism through which the EV71 2C protein induces A3G degradation. The results showed that Baf-A1 and thapsigargin could strongly impair 2C-mediated A3G degradation (Figure 7C), while MG132 could not (Figure 7B).

Co-immunoprecipitation assays showed that A3G could be precipitated with HA-tagged 2C in the presence of the Baf-A1 inhibitor, which protects A3G from degradation, while no A3G was pulled down in the negative control group; these results indicated a specific interaction between A3G and the 2C protein (Figure 7D). The autophagy–lysosome pathway also involves the ubiquitination of the target protein, so we investigated whether 2C-mediated autophagy correlates with A3G polyubiquitination. We found that 2C induces the polyubiquitination of A3G but not 2C itself (Figure 7E). Confocal assays also showed that A3G and 2C co-localized in the cytoplasm together (Figure 7F). Moreover, we observed that EV71 2C induced autophagic puncta as reported in previous studies (34,35). p62/SQSMT1 is a ubiquitin-binding autophagic adaptor that plays an essential role in autophagosome formation. Importantly, we observed that both p62 and A3G localized to the cytoplasm in the absence of 2C. However, in the presence of 2C, p62 and A3G co-localized to the puncta induced by 2C (Figure 7G). Therefore, we concluded that EV71 2C induced A3G degradation through the autophagy–lysosome pathway. Surprisingly, compared to WT EV71, an EV71 infectious clone with 2C deletion lost its infectivity ability, even in non-A3G-expressing HEK293T cells (Supplementary 5A), indicating that EV71 2C is essential for EV71 viral replication. In addition, 2C induced the degradation of other APOBEC3 proteins but not A3H-II (Supplementary 5B).

### The functional domain of EV71 2C is required for A3G degradation

**Figure 8.**
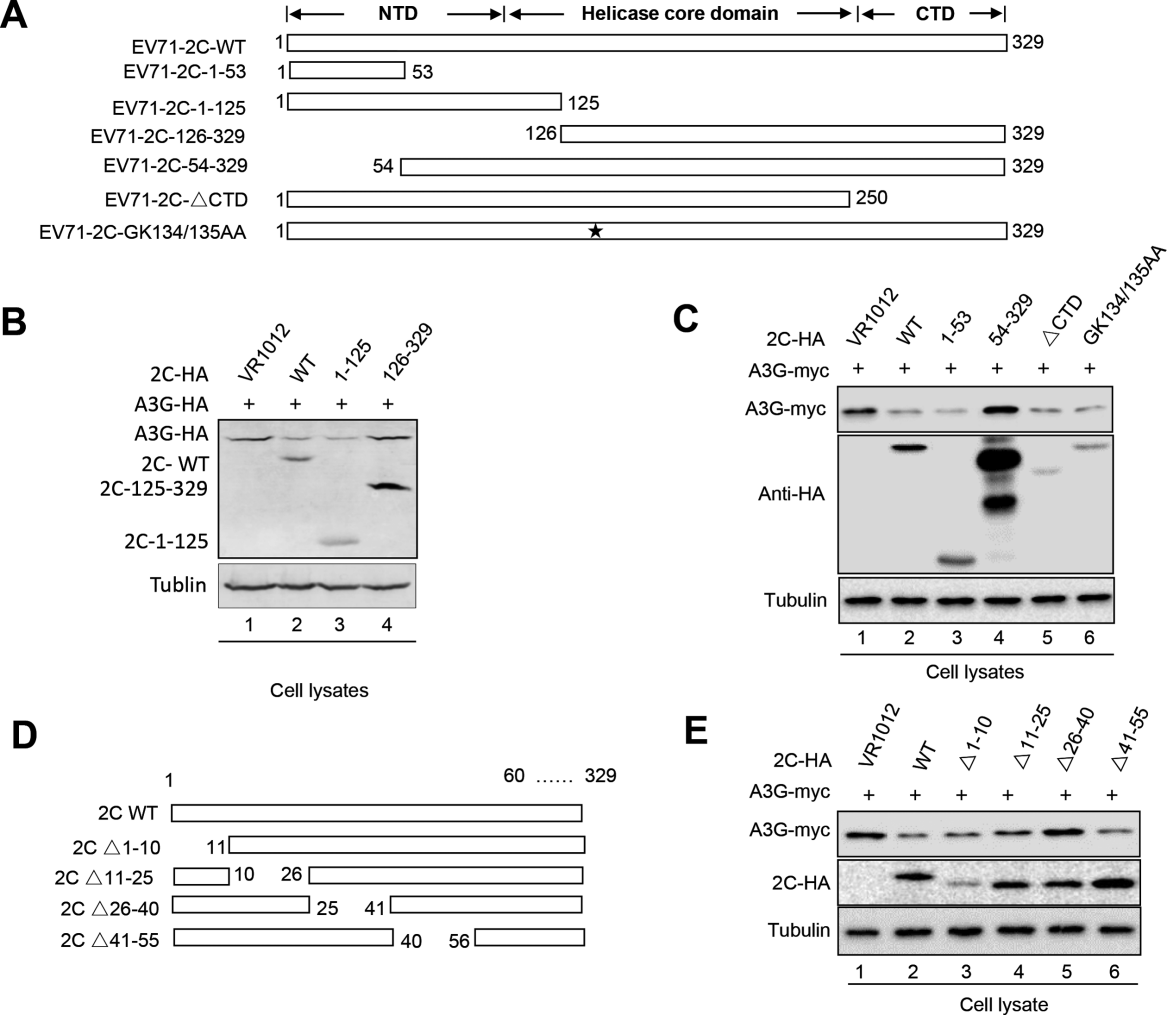
The functional domain of EV71 2C is required for A3G degradation. (**A**) EV71 2C mutant construction. (**B** and **C**) The amino-terminal domain but not the carboxy-terminal domain of EV71 2C is required for 2C-mediated degradation. HEK293T cells were co-transfected as indicated and then harvested for immunoblotting analysis at 48 h post-transfection. (**D**) Truncated mutants in the amino-terminal domain of EV712C were constructed. (**E**) Amino acids 26–40 were required for the 2C-mediated degradation of A3G.

2C might contain three structurally independent domains: the N-terminal domain (NTD), the middle helicase core (HC) domain and the C-terminal domain (CTD) (59,60). To identify the functional domain in EV71 2C that is required for A3G degradation, truncated 2C mutants were constructed based on computation-generated structural modeling of 2C (Figure 8A). Western blotting assays showed that amino acids 1–125 at the N-terminus of 2C were sufficient for A3G degradation, while amino acids 126–329 of 2C were not required for A3G degradation (Figure 8B, lanes 3 and 4). Moreover, GK134/135AA substitutions, which disrupt the ATPase and helicase activities of 2C, and a CTD mutant, which completely lacks RNA chaperoning activities, did not inhibit A3G degradation (Figure 8C, lanes 4 and 5). The truncated mutant 54–329 lost the ability to degrade A3G, while amino acids 1–53 induced A3G degradation, indicating that amino acids 1–53 are sufficient for A3G degradation (Figure 8C, lane 3). Further investigation of a series of short truncated 2C mutants showed that amino acids 26–40 of 2C are critical for A3G degradation (Figure 8E, lanes 3 and 5). We have thus determined the functional domains of 2C that are required for A3G degradation, which will be useful for the development of antiviral drugs targeting the 2C protein.

To examine whether the functional domains of EV71 2C responsible for A3G degradation are also required for 2C-induced autophagy, the LC3 I/LC3 II ratio and autophagic puncta were investigated. Amino acids 1–53 and 1–125 could increase the expression of LC II and induce autophagic flux similar to WT 2C, but amino acids 54–329 and 126–329 could not (Supplementary Figures S6A and S6B). Further investigation determined that A3G degradation was abolished by mutant Δ26–40, as well as ability to increase LC3 II expression and autophagic flux (Supplementary Figure S7). These data further demonstrated that EV71 2C degraded A3G via the autophagy–lysosome pathway.

2C proteins from other picornaviruses, such as enterovirus D68, CA6 and CA16, also showed ability similar to EV71 2C to degrade A3G (Supplementary Figure S8A). Moreover, these 2C proteins also increased the ratio of LC II to LC I and produced autophagic puncta (Supplementary Figures S8B and S8C), indicating that the autophagy-dependent degradation of A3G mediated by EV 2C proteins might be a common mechanism through which EV71 antagonizes the host restriction factor A3G.

## DISCUSSION

A3G potently inhibits diverse retroviruses, DNA viruses, retrotransposons and so on (17–23,28,58). However, whether A3G inhibits the replication of EV71, a human enterovirus species A of the genus *Enterovirus* within the family *Picornaviridae*, and the mechanism through which A3G inhibits EV71 are unknown thus far. In this study, we demonstrated for the first time that A3G could inhibit EV71 virus replication, and this inhibition was not associated with its cytidine deaminase activity (Figures 1, 2 and 3). The clinical manifestations caused by EV71 are usually skin vesicular lesions, oral ulcers and even severe neurological symptoms; in addition, EV71 induced severe lesions in cardiac and skeletal hind-limb muscles in neonatal mice, indicating the tissue tropism of EV71 (,^36^,^61^), which is in agreement with a previous report that skeletal muscles contain lower mRNA levels of most APOBEC3 proteins (62). Therefore, A3G-mediated EV71 infection inhibition might be closely associated with its clinical manifestation. Interestingly, although A3G can inhibit EV71 replication, it could be packaged into EV71 virions if detected (Figure 1A, lower panel), which is different from the inhibitory effects of A3G on HIV-1 where A3G incorporation into Vif-deficient virions is essential for its antiviral function (46,63).

The molecular mechanisms of the anti-viral activity of A3G have been attributed primarily to biochemical characteristics common to APOBEC3 proteins: catalysing cytidine deamination in single-stranded DNA (ssDNA) and nucleic acid-binding capability that is specific to ssDNA or ssRNA (58,63–68). A3G mutants impede RNA binding but still maintain DNA mutation activity to alleviate deamination-independent restriction, indicating a direct correlation between A3G’s capacity to bind RNA and its anti-viral infectivity in a deamination-independent manner (58,64,69–71). Since A3G C291S with a mutation at the C-terminus of A3G that inactivates cytidine deaminase maintained the ability to inhibit EV71 replication (Figure 1), we deduced that the nucleic acid binding property of A3G (26,69,72) might play an important role in the restriction of EV71 replication by A3G. Consistent with our hypothesis, A3G more strongly binds to the 5′UTR of EV71 than the positive control PCBP1, which forms nucleoprotein complexes with the 5′UTR and is required for the synthesis of viral proteins and viral RNA (10–13,34,73). Indeed, competitive co-IP and pull-down assays showed that A3G competitively binds to the 5′UTR with PCBP1 (Figure 4), resulting in the attenuation of VP1 protein expression and production of the EV71 virus. Moreover, the carboxy-terminus of A3G as well as L123A, Y124A and Y125A mutants in 123–127 motif, which lost the ability to bind nucleic acid, could not inhibit EV71 replication (Figure 5). Loop I, identified as the binding domain in the 5′UTR required for A3G binding, is also essential for PCBP1 binding (11), further supporting our hypothesis that nucleic acid binding activity contributes to anti-EV71 activity (Figure 6).

APOBEC3 proteins form a multigene family of cytidine deaminases with diverse inhibitory activities against viruses and retrotransposons. A3A is known to potently inhibit the replication of the parvovirus adeno-associated virus (AAV), which is a single-stranded DNA virus, and this restriction appears to be independent of catalytic activity (74,75). In addition to A3G, we also examined the restriction of other APOBEC3 proteins on EV71 viral replication and 5′UTR activity; we found potent inhibitory activities of A3A, A3D and A3F toward the EV71 5′UTR and viral replication (Supplementary Figures S3 and S4). In contrast, EV71 infection reduces the expression of some APOBEC3 proteins, such as A3B and A3D. However, the mechanism of this reduction should be investigated further due to the distinct functions of the APOBEC3 family members.

Since HIV-1 has developed a counter strategy of encoding the Vif protein to help shield the virus from APOBEC3 function, we propose that EV71 also possesses a special weapon to antagonize the restriction of A3G. Here, we identified EV71 2C as an A3G antagonist protein. We first found that EV71 could reduce the expression of A3G upon EV71 proliferation (Figures 1 and 2). Further analysis confirmed that the EV71 non-structural 2C protein could downregulate A3G at the protein level (Figure 7). Previous studies reported that EV71 2C possesses multiple functions; for example, it can act as an RNA helicase that 3′-5′ unwinds RNA helices in an adenosine triphosphate (ATP)-dependent manner and can inhibit NF-κB activation by binding to RelA (p65) (59,60). Here, 2C antagonized A3G inhibition through an autophagy–lysosome degradation pathway involving A3G polyubiquitination to facilitate EV71 viral replication; the proteasome pathway was not involved in this process (Figure 7). Coincidently, EV71-induced autophagy has been reported to increase viral replication and pathogenesis (34,35). Our data that EV71 2C utilize the autophagy–lysosome pathway to specifically degrade A3G are significant for better understanding the mechanism of viral escape from host resistance.

In addition to EV71, we also demonstrated that A3G could inhibit CA16 replication (Supplementary Figure S1). Moreover, A3G also suppresses the 5′UTR activities of CA16 and PV due to higher homology: 84.09% for EV71 and CA16 and 70.71% for EV71 and PV according to full-length alignment analyses. However, whether A3G or other APOBEC3 proteins restrict other enteroviruses, such as CA6 or CVB3, or even other single-stranded RNA viruses that require PCBP proteins for viral replication, such as PV (12), is worth investigation in the future. Our results broaden our knowledge of host-virus interactions and represent one of the most interesting developments in host restriction factors and single-stranded positive RNA viruses. Considering the broad-spectrum and efficient anti-viral activity of A3G, developing anti-viral inhibitors that avoid A3G degradation or competitively and specifically bind to viral RNAs, such as A3G, would be a good strategy for EV71 therapy.

## Supplementary Material

Supplementary Data
