# Supplementary material for: Enterovirus 71 antagonizes the inhibition of the host intrinsic antiviral factor A3G
Source: Nucleic Acids Res. 2018 Sep 21;46(21):11514–27. doi: 10.1093/nar/gky840 (PMC6265463; doi:10.1093/nar/gky840)
Supplement: Supplementary Data [file gky840_supplemental_files.pdf]

Enterovirus 71 antagonizes the inhibition of the host intrinsic antiviral factor A3G

Zhaolong Li[1], Shanshan Ning[1], Xing Su[1], Xin Liu[1], Hong Wang[1], Yue Liu[1], Wenwen Zheng[1], Baisong Zheng[1], Xiao-Fang Yu[1,2] and Wenyan Zhang[1]*

[1]The First Hospital of Jilin University, Institute of Virology and AIDS Research, Changchun 130021, P. R. China; [2]Cancer Institute (Key Laboratory of Cancer Prevention and Intervention, Ministry of Education), Second Affiliated Hospital, School of Medicine, Zhejiang University, Hangzhou, 310058, P. R. China.

***Corresponding author:**

Institute of Virology and AIDS Research, The First Hospital of Jilin University, No. 519 East Minzhu Avenue, Changchun 130021, P. R. China. Tel.: (86) 431-88782148, Fax: (86) 431-85654528. Email: zhangwenyan@jlu.edu.cn

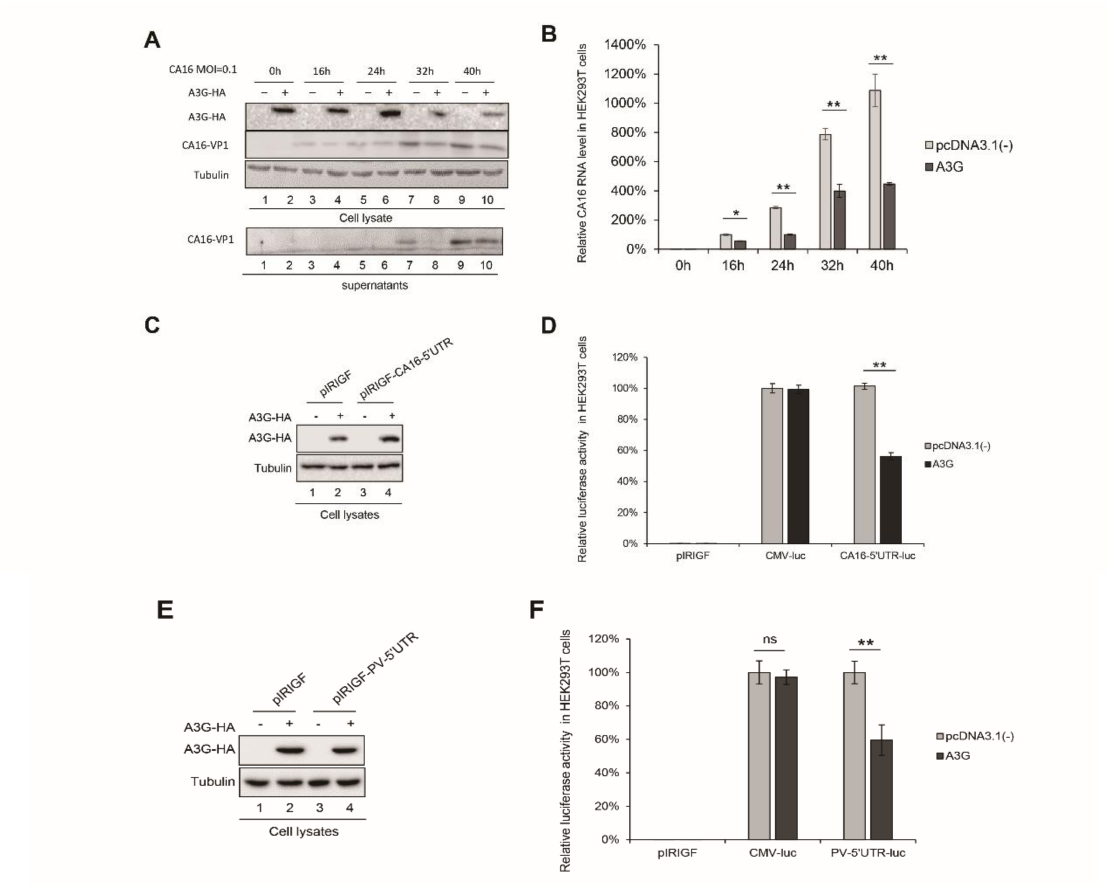

Supplementary Fig. 1. A3G inhibits CA16 replication. HEK293T cells were transfected with pcDNA3.1 or A3G, then infected with DMEM medium or CA16 virus at 0.1 MOI 24 h post transfection. Cells were harvested at indicated time point and loaded for immunoblotting analysis or RNA extraction for RT-qPCR detection. (A) A3G inhibited the expression of CA16 VP1 protein. (B) A3G inhibited synthesis of CA16 viral RNA. GAPDH was used as a control. CA16 RNA level of cells transfected with pcDNA3.1 at 16 h was set as 100%. (C and E) Expression of A3G was detected by immunoblotting analysis. Effects of A3G on CA16 (D) or Polivirus (F) 5'UTR activity. Luciferase activity downstream of CMV in the absence of A3G was set as 100%. (B, D and F) Results are means with SD from at least three independent experiments. Asterisks indicate statistically significant differences between groups as assessed by the Student's t-test (*$P < 0.05$, **$P < 0.01$, ***$P < 0.001$).

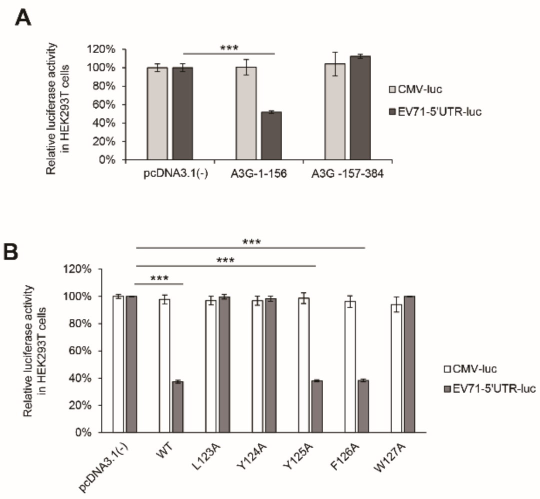

Supplementary Fig. 2. (A) The amino-terminal of A3G could specifically inhibit EV71 5'UTR activity but the carboxy-terminal couldn't. (B) A3G mutants L123A, Y124A and W127A couldn’t inhibit 5'UTR activity.

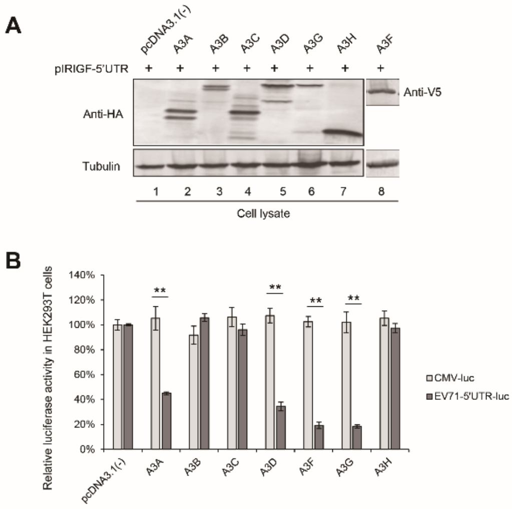

Supplementary Fig. 3. Effect of APOBEC3 proteins on EV71 5’UTR activity. (A) Expression of APOBEC3 proteins. (B) Effect of APOBEC3 proteins on translational activity of EV71 5’UTR. Luciferase activity downstream of CMV in the absence of A3G was set as 100%. Results are means with SD from at least three independent experiments. Asterisks indicate statistically significant differences between groups as assessed by the Student's t-test (*$P < 0.05$, **$P < 0.01$, ***$P < 0.001$).

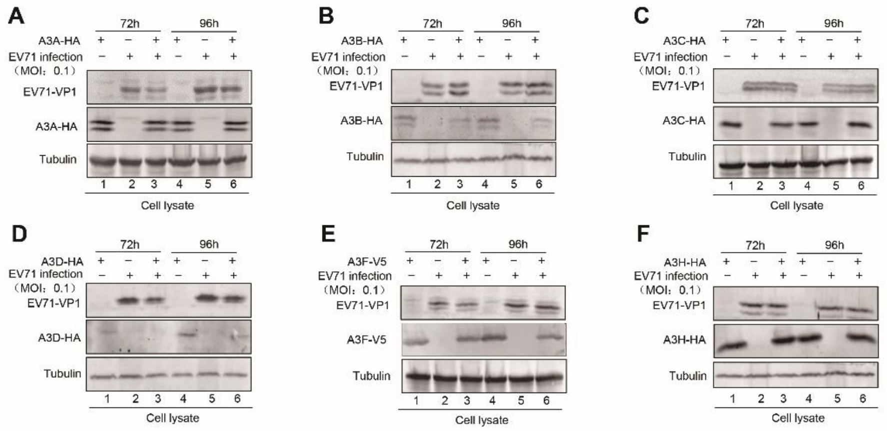

Supplementary Fig. 4. The effect of APOBEC3 proteins on EV71 viral replication. HEK293T cells were co-transfected with A3A-HA (A), A3B-HA (B), A3C-HA (C), A3D-HA (D), A3F-V5 (E), A3H-II-HA (F) or negative control vector as indicated. 24 h post transfection, cells were infected with EV71 virus at 0.1 MOI. The cells were harvested at 72 h and 96 h and loaded for immunoblotting analysis using an anti-VP1, anti-HA, anti-V5 or anti-tubulin antibody.

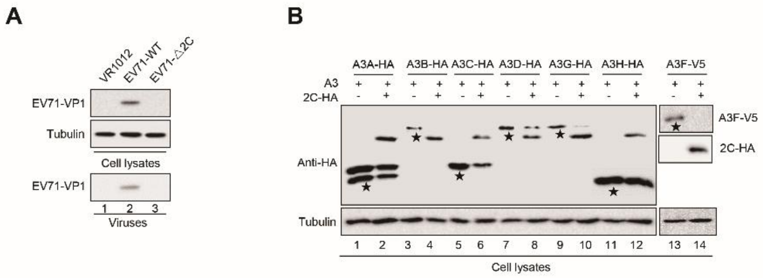

Supplementary Fig. 5. (A) Replication ability of WT EV71 or EV71 deleted for 2C infectious clone in HEK293T cells. (B) The effect of EV71 2C on other APOBEC3 proteins by immunoblotting analysis.

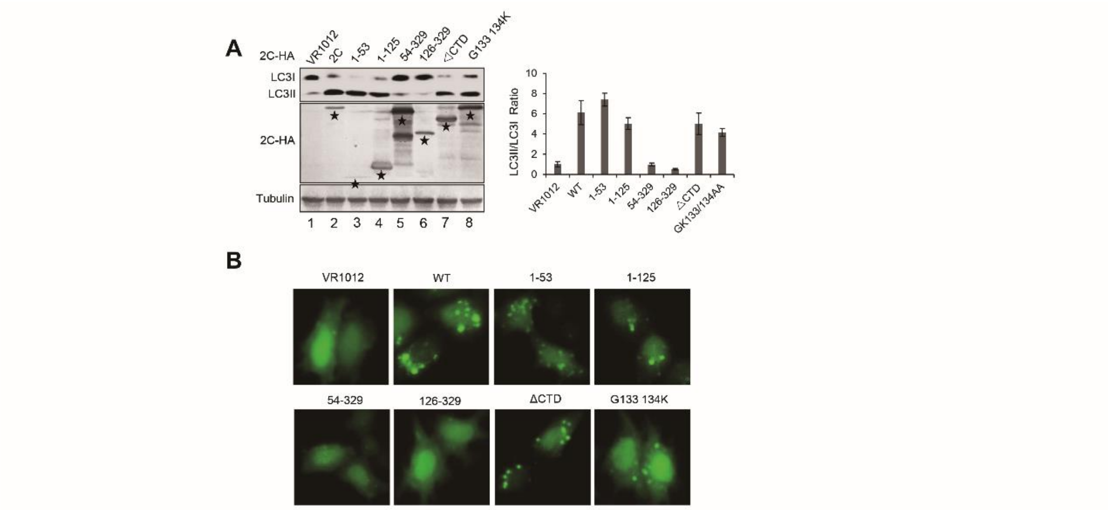

Supplementary Fig. 6. Functional domain in 2C required for A3G degradation was also responsible for autophage function of 2C. (A)The effect of 2C or its mutants on LC3 expression by immnuoblotting analysis. (B) The effect of 2C or its mutants on the formation of autophagic puncta. The cells were imaged by confocal microscopy.

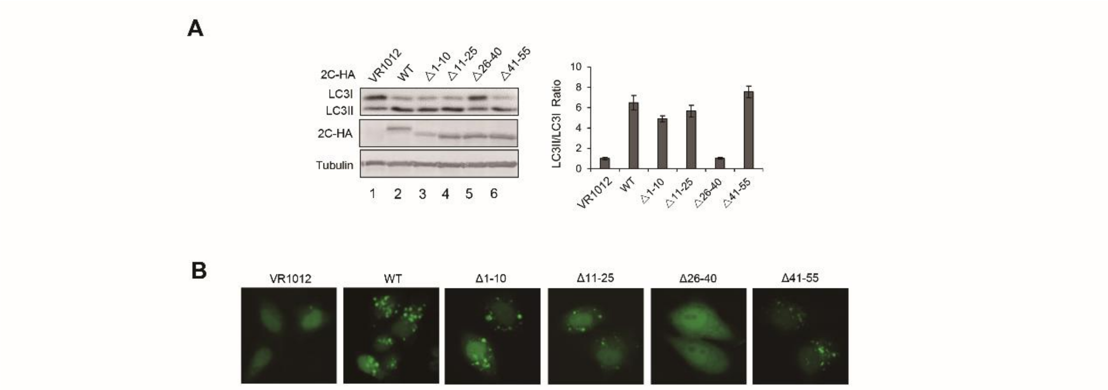

Supplementary Fig. 7. Amino acid 25–40 in 2C required for A3G degradation is also important for 2C induced autophage. (A) Δ26-40 mutant couldn't increase LC3 II expression by immnuoblotting analysis. (B) Δ26-40 mutant lost the ability to produce the autophagic puncta by confocal microscopy analysis.

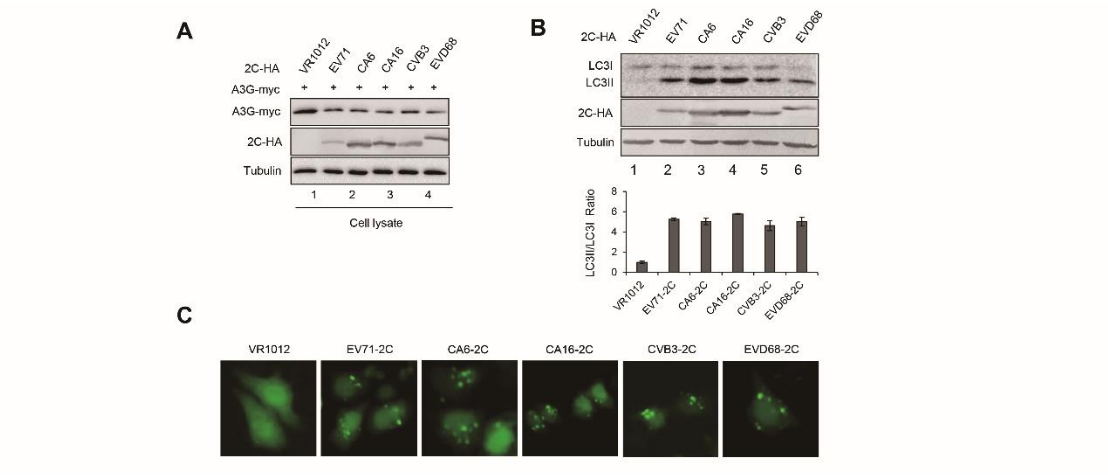

Supplementary Fig. 8. Function analysis of EV D68, CVA6 and CVA16 2C proteins. (A) EV D68, CVA6 and CVA16 2C proteins all induced A3G degradation by immnuoblotting analysis. (B) EV D68, CVA6 and CVA16 2C proteins all induced autophage. LC3 I and II expression were analyzed by immunoblotting. (C) EV D68, CVA6 and CVA16 2C induced autophagic puncta by confocal microscopy analysis.
